# Risk stratification in childhood hypertrophic cardiomyopathy

**DOI:** 10.21542/gcsp.2018.24

**Published:** 2018-08-12

**Authors:** Gabrielle Norrish, Juan Pablo Kaski

**Affiliations:** Centre for Inherited Cardiovascular Diseases, Great Ormond Street Hospital, London, UK; University College London Institute of Cardiovascular Science, London, UK

## Introduction

The true prevalence of hypertrophic cardiomyopathy (HCM) in childhood is unknown, but population-based studies have reported an annual incidence between 0.24–0.47 per 100,000 children^1–3^. The aetiology of disease is more heterogeneous than that seen in adult populations, with up to 30% of patients having an inborn error of metabolism, malformation syndrome or neuromuscular syndrome^4,5^. However, as in adults, most cases are caused by mutations in the cardiac sarcomere protein genes, even in young children^6,7^. The long-term outcome of childhood HCM is highly variable and has been shown to depend partly on the age of presentation and underlying aetiology^4,8,9^. Outside of infancy, the most frequent cause of mortality is sudden cardiac death (SCD), and one of the greatest challenges in managing young patients with HCM is identifying those at greatest risk of an arrhythmic event.

HCM has been reported as the most common cause of SCD in young athletes^10^, but published estimates of the rate of SCD in childhood HCM have varied widely. Initial reports from small, highly selected populations suggested a very high mortality secondary to SCD, with reported rates of up to 7% per year^11^. Yet, reflecting what has been seen in adult practice, the reported SCD rates have fallen over time. The most recent population-based studies report an annual SCD rate of 1–2% per year^5,8^, which, although lower than previously thought, is still higher than the SCD rates reported in adults. There is therefore a subset of children who would benefit from preventative implantable cardioverter defibrillator (ICD) therapy. ICDs have been shown to be effective at terminating malignant arrhythmias in childhood HCM, but these young patients experience a higher rate of complications compared to adults. There is a need to robustly identify those patients who are most likely to benefit from this intervention.

## Risk factors for SCD in childhood HCM

Risk factors for SCD in adult HCM include prior ventricular fibrillation (VF) or sustained ventricular tachycardia (VT); family history of sudden cardiac death; unexplained syncope; non-sustained ventricular tachycardia (NSVT); maximal left ventricular wall thickness >30 mm; left atrial dilatation; and left ventricular outflow tract obstruction. These conventional clinical risk factors are used for risk stratification of SCD in adult patients.

Although a number of potential risk factors for SCD in childhood HCM have been reported in the literature over the past 30 years, the lack of consistent definitions and well-designed, large population studies means that the evidence for individual risk factors is not robust. A recent systematic review and meta-analysis of clinical risk factors for SCD in childhood HCM^12^ identified twenty-five studies which explored the role of twenty- three separate clinical risk factors. The majority of studies were retrospective, small (all but 3 studies had less than 150 participants) and described heterogeneous populations. Four clinical risk factors were identified as being ‘major risk factors’ and likely to be associated with SCD in childhood HCM (Table 1): previous aborted cardiac arrest or sustained VT; unexplained syncope; NSVT; and extreme left ventricular hypertrophy. Left atrial diameter did not meet the major risk factor criteria, but was associated with SCD in two out of three studies. ‘Minor risk factors’ included a family history of SCD, gender, age, symptoms, electrocardiograph findings, blood pressure response to exercise and left ventricular outflow tract obstruction. The evidence for the role of individual clinical risk factors is summarised below and in Table 2.

**Table 1:** Risk factors for sudden cardiac death in childhood hypertrophic cardiomyopathy. Adapted from Norrish et al.

‘Major’ clinical risk factor^a^	Hazard ratio (95% confidence interval)
Previous aborted cardiac event	5.4 (3.67–7.95), *p* < 0.001
Non-sustained ventricular tachycardia	2.13 (1.21–3.74), *p* = 0.009
Unexplained syncope	1.89 (0.69–5.16), *p* = 0.22
Extreme left ventricular hypertrophy^b^	1.8 (0.75–4.32), *p* = 0.19
**‘Minor’ risk factor^c^**	
Left atrial diameter, Family history SCD, Gender, Age, Symptoms, ECG changes, Abnormal blood pressure response to exercise, LVOTO	

**Notes.**

a ‘Major’ risk factor defined as being investigated in at least 4 studies and significantly associated with SCD in ≥ 2 statistical analysis.

b Maximum LV thickness >30mm, or Z-score >6.

c ‘Minor’ risk factor defined as being associated with SCD in 1 analysis.

### Previous adverse cardiac event

**Figure 1.**
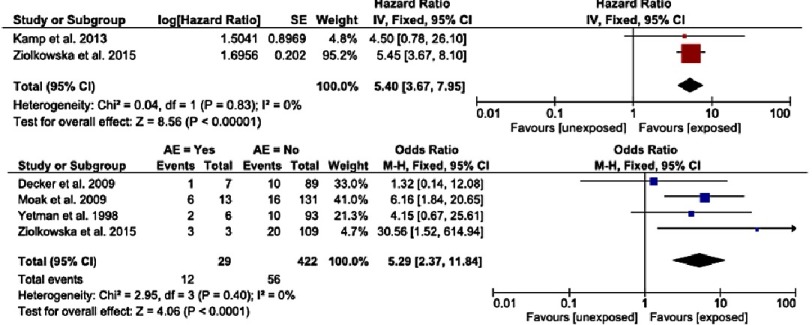
a) Hazard ratios for sudden cardiac death or cardiovascular death for previous adverse event. The size of the square corresponds with the number of patients in the study. The bars represent the upper and lower 95% CI. Hazard ratios with CI >1 indicate a significant association with sudden cardiac death. b) Odds ratios for sudden cardiac death or cardiovascular death for previous adverse event. The size of the square corresponds with the number of patients in the study. The bars represent the upper and lower 95% CI. Odds ratios with CI >1 indicate a significant association with sudden cardiac death. Reproduced from Norrish et al.^12^

A history of a previous aborted cardiac arrest due to VT or VF, or sustained VT causing haemodynamic compromise is recognised to be a risk factor for SCD in childhood HCM, with a hazard ratio of 5.4 (95% CI [3.68–7.95], *p* < 0.001)^12–17^(Figure 1). In recognition of this, both the European and North American guidelines recommend the implantation of an ICD for secondary prevention if there has been a previous adverse event^18,19^ (Figure 5).

**Table 2:** Summary of evidence for ‘Minor’ risk factors for sudden cardiac death in childhood hypertrophic cardiomyopathy.

Clinical risk factors	Summary of evidence
Gender	No studies have reported a significant association between SCD and gender
Age	Presentation in infancy is associated with an increased risk of mortality secondary to congestive cardiac failure^5,8^. Outside infancy the majority of studies found no association with age and risk of SCD.
Symptoms	A wide range of symptoms can be seen in childhood HCM and the role of symptoms in risk stratification for SCD has not been systematically assessed.
Family history of SCD	Only 1 paediatric study has reported a significant association with SCD^15^.
ECG changes	QTc dispersion has been reported to be associated with SCD in 2 studies^13,17^.
	Other ECG parameters that have been analysed include RS sum^23^ and heart rate variability^31,32^.
Abnormal BP response to exercise	No studies have reported a significant association with SCD
Left atrial enlargement	Increased left atrial size was associated with an increased risk of SCD in two studies^17,24,25^
Left ventricular outflow tract obstruction	Only 1 paediatric study reported an increased risk of SCD with increasing LVOT gradient^17^. A gradient above 30mmHg was not predictive of SCD in this study.
Restrictive physiology	Echocardiographic markers for restrictive physiology may increase the risk for SCD^17,24,25^
Late Gadolinium enhancement (LGE) on cardiac Magnetic Resonance	The presence of LGE has been shown to be associated with increased LV wall thickness/mass^33,34^. An independent role for LGE in predicting SCD in childhood HCM has not been show.

### Unexplained syncope

**Figure 2.**
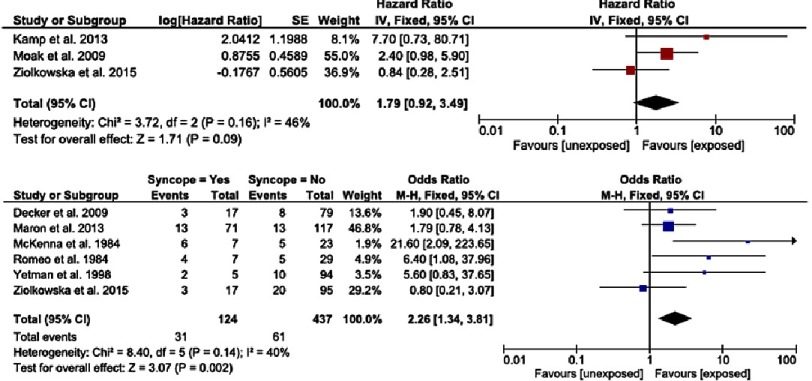
a) Hazard ratios for sudden cardiac death or cardiovascular death for unexplained syncope. b) Odds ratio for sudden cardiac death or cardiovascular death for unexplained syncope. Reproduced from Norrish et al. ^12^.

Syncope that is unexplained after investigation has been evaluated in several studies^13,15–17,20,21^ and shown to be associated with an increased with of SCD in childhood HCM. The reported hazard ratio is 1.89 (95% CI [0.69–5.16], *p* = 0.22) and odds ratio estimate is 2.64 (96% CI [1.21–5.79], *p* = 0.02) (Figure 2). In adults with HCM, syncope occurring within the last 6 months may be more predictive of SCD compared to a more distant syncopal event; the importance of timing of syncopal events has not been explored in childhood HCM.

**Figure 3.**
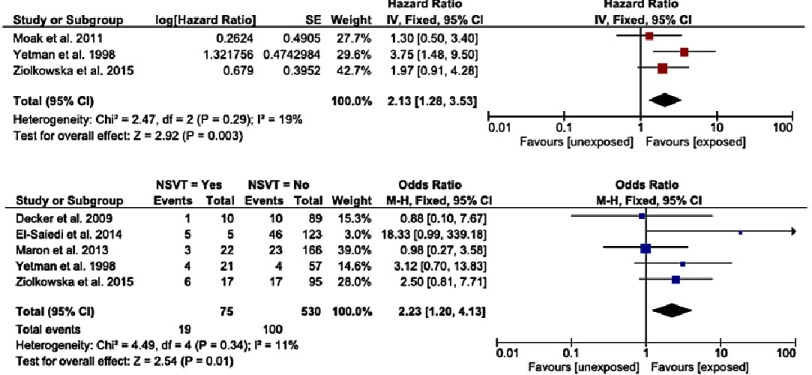
a) Hazard ratios for sudden cardiac death or cardiovascular death for non-sustained VT. b) Odds ratio for sudden cardiac death or cardiovascular death for non-sustained VT. Reproduced from Norrish et al.^12^

### NSVT

Non-sustained ventricular tachycardia (NSVT) is defined as three or more consecutive ventricular beats occurring at a rate of greater than 120 beats/min with a duration of less than 30 seconds on ambulatory ECG recordings^18^. The prevalence of NSVT during ambulatory ECG monitoring in childhood HCM is unknown, but it has been shown to be an independent predictor of SCD in childhood HCM with a combined hazard ratio of 2.13 (95% CI [1.21–3.74], *p* = 0.0009) (Figure 3)^13,14,16,17,21,22^.

### Maximum left ventricular wall thickness

**Figure 4.**
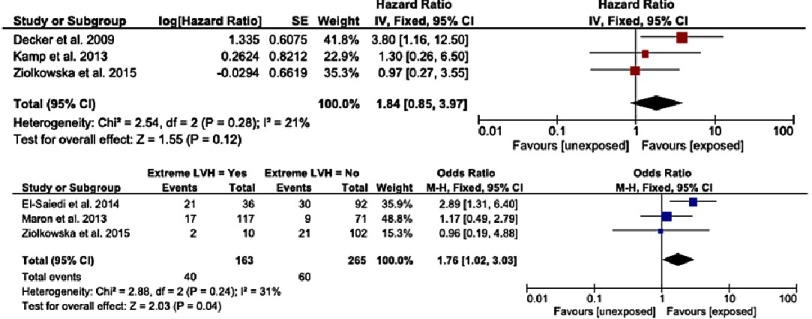
a) Hazard ratios for sudden cardiac death or cardiovascular death for extreme LVH. b) Odds ratio for sudden cardiac death or cardiovascular death for extreme LVH. Reproduced from Norrish et al.^12^

The association between SCD and severity or extent of left ventricular hypertrophy (LVH) has been investigated in multiple studies^4,13–17,21–23^, several of which have reported a significant association^16,21,23^ (Figure 4). However, there is significant variability in the measurement of LVH. There is currently no consensus on the most clinically important measure of LVH for risk stratification. The ESC guidelines include severe LVH (maximum LV wall thickness ≥30 mm or Z-score ≥6) as a risk factor for SCD, but, importantly, only 1 study has shown a significantly increased risk of SCD using this definition.

**Figure 5.**
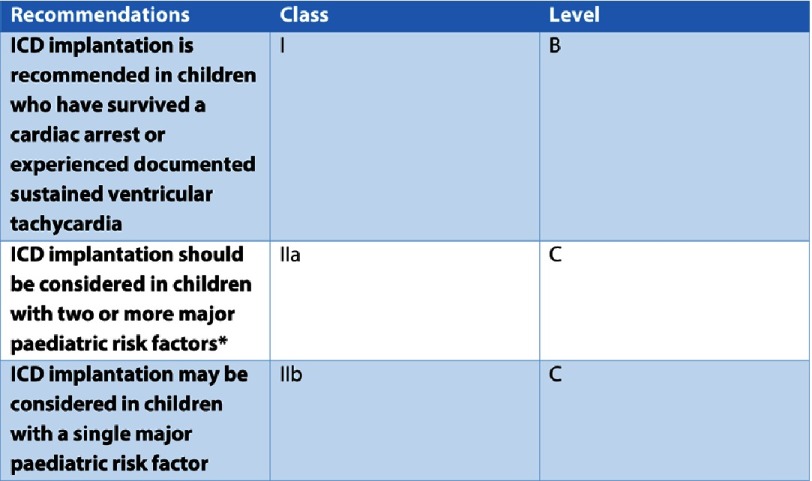
European Society of Cardiology recommendations for implantation of cardioverter defibrillators in children. *Major paediatric risk factors: Maximum left ventricular wall thickness ≥30mmor a Z-score ≥6, unexplained syncope, non-sustained ventricular tachycardia (≥3 consecutive ventricular beats at ≥120 BPM lasting, 30 seconds), family history of SCD (one or more first-degree relatives with SCD aged, 40 years with or without the diagnosis of HCM, or SCD in a first-degree relative at any age with an established diagnosis of HCM).

### Family history of SCD

A family history of SCD is defined as the sudden death of a first-degree relative <40 years of age with or without a known diagnosis of HCM, or SCD in a first-degree relative at any age with an established diagnosis of HCM^18^. It is included as a risk factor for SCD in the ESC guidelines, but, despite multiple studies investigating this risk factor^4,13–16,22^, only one paediatric study has reported a significant association^15^. This is in contrast to adult practice, where multiple studies have shown an increased risk of SCD in the presence of a family history. Possible explanations for this difference include a higher prevalence of *de novo* mutations in childhood HCM, a lower proportion of sarcomeric disease and insufficient reporting of family history in published studies.

### Left ventricular outflow tract obstruction

LVOT obstruction is conventionally accepted as a risk factor for SCD in adult HCM, but, although it has been evaluated in several paediatric studies^8,13,14,17,22^, only one reported an increased risk of SCD with increasing LVOT gradient^17^. A gradient above 30mmHg was not predictive of SCD in this study.

### Left atrial enlargement

Increased left atrial size was associated with an increased risk of SCD in two studies^17,24,25^ and is likely to be important for risk stratification in childhood HCM.

### Abnormal blood pressure response to exercise

An abnormal blood pressure response to exercise (ABPRE) is defined as the failure of systolic blood pressure (BP) to rise by ≥ 20mmHg (flat blood pressure response) or a fall in blood pressure during exercise (hypotensive blood pressure response). Patients <40 years with an ABPRE have been reported to have a higher risk of SCD^26^, but no significant association has been found in paediatric studies^14–17^.

### Current approach to risk stratification in childhood HCM

Not surprisingly, given the sparsity of evidence, the current European and American guidelines^18,19^ contain only short sections on risk stratification for SCD in childhood HCM. Both guidelines recommend the use of four clinical risk factors to predict SCD (maximum LV wall thickness ≥ 30 mm or Z score ≥6; unexplained syncope; NSVT; or family history of SCD). The European guidelines recommend the implantation of an ICD for primary prevention in those with two or more risk factors^18^, whilst the North American guidelines state an ICD should be considered in the presence of a single risk factor^19^ (Figure 1). As discussed above, the evidence supporting the use of some of these risk factors to predict SCD in childhood HCM is not robust. Aside from the clinical risk factors chosen, this approach to risk stratification provides relative rather than absolute risks for non-homogeneous groups, and necessarily uses binary cut-offs for the purpose of risk stratification, the clinical validity of which may be questioned. Moreover, the simple summation of risk factors in adult HCM has been shown to have a low predictive power for SCD, leading to unnecessary ICD implantation in some patients^27^. This is a particular concern for young patients who are at increased risk of complications from ICD.

To overcome some of these limitations, the European Society of Cardiology has more recently endorsed the use of a SCD risk-prediction model (HCM Risk-SCD)^18,28^ for adults with HCM. This model provides an individualised estimate for 5-year SCD risk utilising clinical predictor variables associated with SCD in multivariable analyses. Independent, external comparison of these two approaches has shown that the HCM-risk SCD model improves the risk stratification of adult patients with HCM^29,30^. However, this model is currently not validated for patients use in patients under the age of 16 years, and although it can be used for patients aged 16-18, years, this group of young people constituted a small proportion of the development cohort (n=82/3675, 2%) and so further evaluation of their risk may be needed.

### Future of risk stratification in childhood HCM

Risk stratification for SCD in adults and children with HCM remains a significant challenge. Our current understanding of the conventional risk factors for SCD in childhood is limited by the lack of consistent definitions and well-designed, large population studies. Moreover, the role of novel potential risk factors for SCD including genetics and advanced imaging techniques have not yet been systematically evaluated. However, risk stratification for patients presenting at a young age has additional challenges due to the heterogeneity of the patient cohort (including age of presentation, symptoms and underlying aetiology) and the effect of somatic growth, which means that a patient’s phenotype may evolve considerably during childhood, with possible important implications for risk stratification.

As childhood HCM is a rare disease and SCD is a rare outcome, the challenge of improving risk stratification in childhood HCM can only be addressed through multi-centre, large-scale, collaborative projects. In response to this, we have established an International Paediatric Hypertrophic Cardiomyopathy Consortium consisting of 38 cardiac centres worldwide caring for paediatric patients with HCM. A cohort of over 1400 patients has been created which will allow for the systematic evaluation of the role of individual risk factors for SCD in childhood HCM with the aim of improving risk stratification for these patients.
